# Immune-related miRNA-mRNA regulation network in the livers of DHAV-3-infected ducklings

**DOI:** 10.1186/s12864-020-6539-7

**Published:** 2020-02-04

**Authors:** Fengyao Wu, Fengying Lu, Xin Fan, Jin Chao, Chuanmin Liu, Qunxing Pan, Huawei Sun, Xiaofei Zhang

**Affiliations:** 1 Institute of Veterinary Medicine, Jiangsu Academy of Agricultural Sciences, Nanjing, Jiangsu Province China; 2 Key Laboratory of Veterinary Biological Engineering and Technology, Ministry of Agriculture, Nanjing, Jiangsu Province China; 3 Academy of Animal Sciences, Tibet Agriculture and Animal Husbandry University, Linzhi, Tibet Province China; 4 College of Animal Science and Technology, Anhui Agricultural University, Hefei, Anhui Province China

**Keywords:** Duck hepatitis a virus type 3, miRNA, Transcriptome, miRNA-mRNA network, Innate immune response, Host-virus interactions

## Abstract

**Background:**

Duck hepatitis A virus type 3 (DHAV-3) is one of the most harmful pathogens in the duck industry. However, the molecular mechanism underlying DHAV-3 infection in ducklings remains poorly understood. To study the genetic regulatory network for miRNA-mRNA and the signaling pathways involved in DHAV-3 infection in ducklings, we conducted global miRNA and mRNA expression profiling of duckling liver tissues infected with lethal DHAV-3 by high-throughput sequencing.

**Results:**

We found 156 differentially expressed miRNAs (DEMs) and 7717 differentially expressed genes (DEGs) in livers of mock-infected and DHAV-3-infected duckling. A total of 19,606 miRNA-mRNA pairs with negatively correlated expression patterns were identified in miRNA-mRNA networks constructed on the basis of these DEMs and DEGs. Moreover, immune-related pathways, including the cytokine-cytokine receptor interaction, apoptosis, Toll-like receptor, Jak-STAT, and RIG-I-like receptor signaling pathway, were significantly enriched through analyzing functions of mRNAs in the network in response to DHAV-3 infection. Furthermore, apl-miR-32-5p, apl-miR-125-5p, apl-miR-128-3p, apl-miR-460-5p, and novel-m0012-3p were identified as potential regulators in the immune-related signaling pathways during DHAV-3 infection. And some host miRNAs were predicted to target the DHAV-3 genome.

**Conclusions:**

This is the first integrated analysis of miRNA and mRNA in DHAV-3-infected ducklings. The results indicated the important roles of miRNAs in regulating immune response genes and revealed the immune related miRNA-mRNA regulation network in the DHAV-3-infected duckling liver. These findings increase our knowledge of the roles of miRNAs and their target genes in DHAV-3 replication and pathogenesis. They also aid in the understanding of host-virus interactions.

## Background

Duck viral hepatitis is a highly fatal, contagious, and rapidly spreading viral infection in young ducklings [1]. At least five viruses can cause this disease. The most widely distributed causative agent is duck hepatitis A virus (DHAV), which is classified as genus *Avihepatovirus* in the family *Picornaviridae* [2]. Based on phylogenetic analysis and virus neutralization tests, DHAV has been classified into DHAV-1, the most widespread serotype [3], DHAV-3, a serotype isolated in Korea and China [4, 5], and DHAV-2, a serotype isolated only in Taiwan [6]. With the widespread use of the officially approved DHAV-1 live vaccine in 2013, a higher rate of DHAV-3 causing DHAV infections has been observed in China [7].

DHAV-3 causes acute hepatitis and induces typical duckling liver lesions, which is characterized by petechial hemorrhages of the liver surface [8]. The results of detecting DHAV-3 loads in the liver exhibited that the virus can replicate rapidly in the liver [9]. The highest viral titer was detected in the liver among all DHAV-3 infected organs in vivo [10]. The liver is a major site for the regulation of immune and inflammatory responses and plays a critical role in defense against invasive pathogens [11, 12]. However, there is little information on miRNA combined with gene expression profiles in duckling liver in response to DHAV-3 infection.

MicroRNAs (miRNAs) are a class of endogenous, small RNAs (21–24 nt) that possess a regulatory role in mRNA translation [13, 14]. They direct the regulation of gene expression by binding to partially complementary target sites in the 3′-untranslated region (3′-UTR) of mRNAs, cause post-transcriptional inhibition and facilitate target gene knockout and degradation [15]. It has been proved that miRNAs have a significant effect on the proliferation of viruses and viral-host interactions [16–18]. For instance, previous reports have shown that several RNA viruses, including dengue virus (DENV), Japanese encephalitis virus (JEV) and West Nile virus (WNV), can be regulated in their replication by host miRNAs [18, 19]. On the other hand, miRNAs can also regulate the expression of host immune-related genes, inhibiting or activating the downstream signaling pathway and mediating the anti-viral immune response [20–24].

**Fig. 1:**
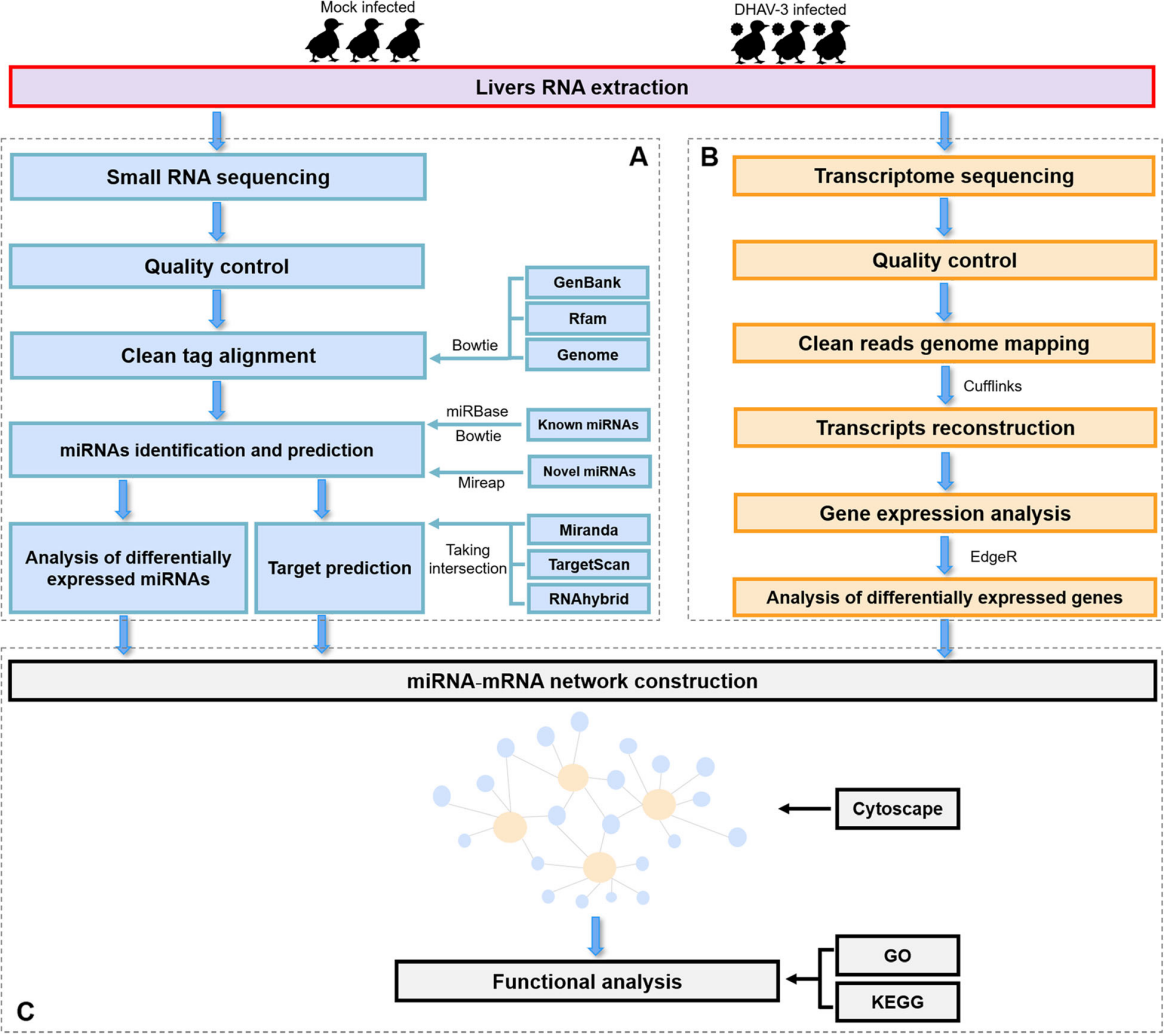
Workflow of analyses and bioinformatic pipelines. **a** Pipeline for identification and analyses of miRNAs. **b** Pipeline for analyses of transcriptome sequencing data. **c** Pipeline for construction of immune-related miRNA-mRNA negative correlation network

Nowadays, high-throughput sequencing is an efficient method to obtain the sum of all miRNAs and mRNA products of a specific tissue or cell in a specific state. After the advent of genome-wide miRNA expression profiles using deep sequencing, several miRNAs studies on ducks have examined their roles in growth, reproduction, and virus infection [25–28]. However, there is no information on miRNA and mRNA expression in duckling liver responding to DHAV-3 infection. We used small RNA sequencing and transcriptome sequencing to provide a global picture of the different mechanisms activated in response to DHAV-3 infection in duckling liver, and the immune related miRNA-mRNA regulation network was preliminarily established (Fig. 1). The results may pave the way to understand the pathogenesis of DHAV-3 and the mechanisms of host-virus interactions.

## Results

### Characterization of DHAV-3 infection in duckling liver

**Fig. 2:**
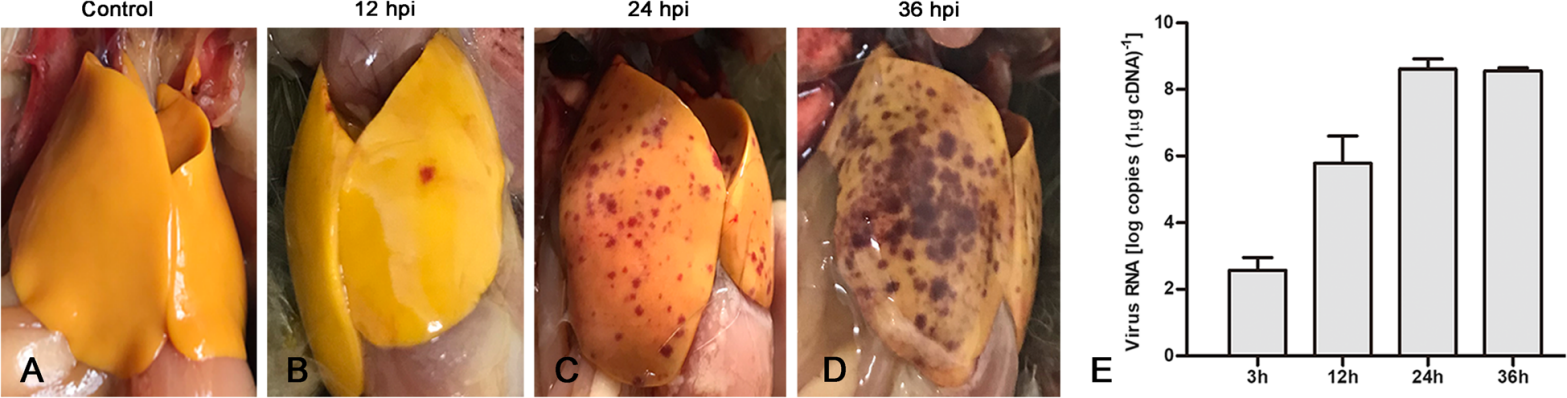
Characterization of DHAV-3 infection in duckling liver. **a** Healthy duckling liver in the control group. **b** Duckling liver at 12 hpi began to have petechial hemorrhages. **c** Duckling liver at 24 hpi is typically enlarged with petechial hemorrhages throughout. **d** Liver of dead duckling at 36 hpi. **e** DHAV-3 replication in the duckling liver at 3, 12, 24 and 36 hpi. The data are expressed as mean ± standard deviation. Three samples were selected for detecting the viral RNA load using the TaqMan qRT-PCR method

One-day-old ducklings infected with virulent DHAV-3 started to appear visible ecchymosis hemorrhages at 12 hpi (Fig. 2b), and exhibited ecchymosis hemorrhages throughout the liver at 24 hpi (Fig. 2c) and at death (Fig. 2d), while no significant histopathological lesions were observed in the liver of the control group (Fig. 2a). Infected ducklings showed typical clinical signs, such as mental depression and anorexia. Mortality occurred within 24–36 hpi. We detected DHAV-3 loads in the livers using TaqMan qRT-PCR. DHAV-3 replicated rapidly in the liver and reached 10^5.78^ copies (1 μg cDNA)^− 1^ at 12 hpi, and 10^8.62^ copies (1 μg cDNA)^− 1^ at 24 hpi (Fig. 2e). On the basis of these results, we chose duckling livers at 24 hpi to perform the RNA-seq analysis.

### Analysis of small RNA libraries

**Fig. 3:**
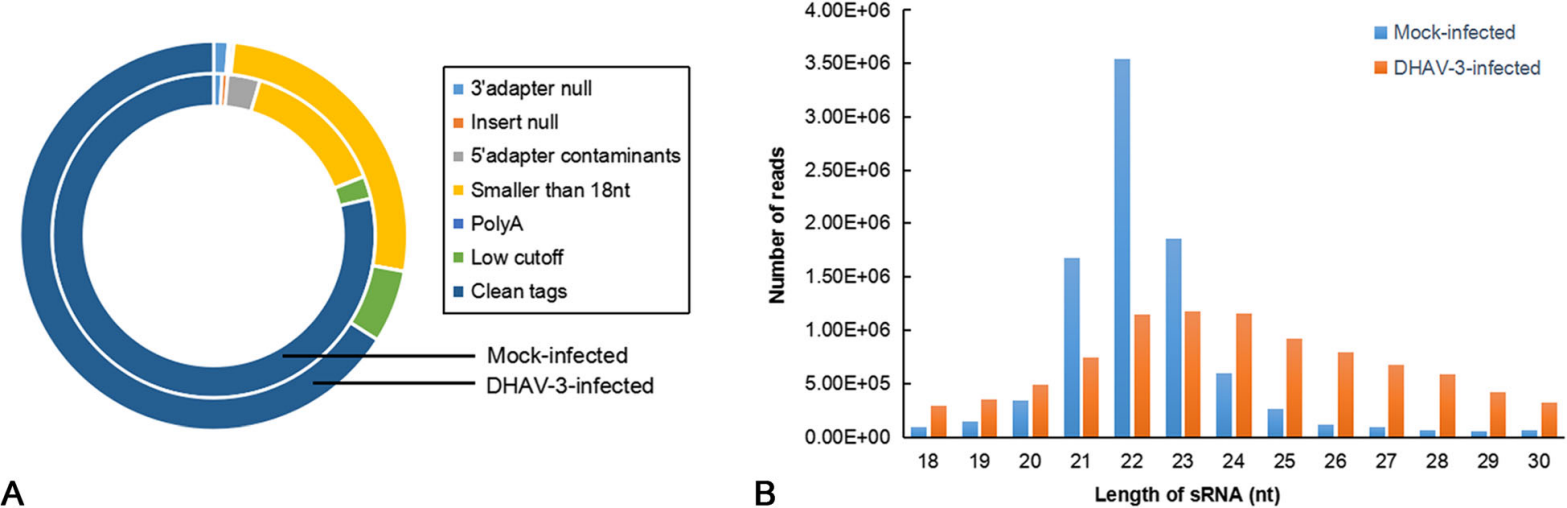
Summary of small RNA sequencing in the mock-infected and DHAV-3-infected duckling liver libraries. **a** Overview of small RNA sequence in the mock-infected and DHAV-3-infected libraries. **b** Length distributions of clean reads in the mock-infected and DHAV-3-infected libraries

To determine the miRNA expression pattern in response to DHAV-3 in the duckling liver, two sRNA libraries from mock-infected and DHAV-3-infected groups were constructed. Following high-throughput sequencing, the total number of raw reads collected from uninfected and infected duckling liver was 11,826,502 and 14,689,775, respectively (Table 1). After removing low-quality sequences, adapter sequences, and short reads smaller than 18 nt, clean reads were obtained (Fig. 3a and Table 1). All of the clean reads were annotated and classified as rRNA, snRNA, snoRNA, snoRNA, tRNA, exon-sense, exon-antisense, intron-sense, intron-antisense, miRNA, and repeats (Table 1). The length distribution of the clean reads was mainly between 21 and 24 nt (Fig. 3b), which is consistent with previous reports [26–28]. These results indicate that miRNAs had been enriched successively from the two libraries.

**Table 1:** Overview of small RNA sequencing in the mock-infected and DHAV-3-infected libraries

Type	Mock-infected duck liver				DHAV-3-infected duck liver			
	Unique	Percent	Total	Percent	Unique	Percent	Total	Percent
Raw reads			11,826,502				14,689,775	
Clean reads	139,965		9,172,408		240,697		9,531,577	
rRNA	17,855	12.76	275,818	3.01	35,351	14.69	2,301,404	24.15
snRNA	462	0.33	2910	0.03	1291	0.54	22,661	0.24
snoRNA	587	0.42	77,678	0.85	670	0.28	11,815	0.12
tRNA	5061	3.62	417,238	4.55	10,225	4.25	1,103,800	11.58
exon_sense	15,717	11.23	43,102	0.47	29,505	12.26	95,225	1.00
exon_antisense	408	0.29	1542	0.02	310	0.13	1949	0.02
intron_sense	5040	3.60	35,317	0.39	4684	1.95	35,333	0.37
intron_antisense	6238	4.46	60,363	0.66	14,173	5.89	1,323,996	13.89
repeat	444	0.32	84,834	0.92	487	0.20	282,012	2.96
miRNA	21,271	15.20	7,281,344	79.39	9365	3.89	1,050,011	11.02
unannotated	66,350	47.40	878,547	9.58	134,035	55.69	3,289,546	34.51

### Identification of known and novel miRNAs

To identify known miRNAs, we aligned sRNA from our libraries to the known mature miRNAs and their precursors in the miRBase 22.0 database. A total of 349 and 291 known miRNAs were identified in the mock- and DHAV-3-infected liver libraries, respectively (Additional file 1). A number of unannotated sRNAs were present in each library (Table 1), and these sRNAs were matched to the duck genome (CAU_duck1.0) for predicting novel miRNA candidates. A total of 109 and 34 novel miRNAs were predicted in the mock-infected and DHAV-3-infected duckling liver libraries using MIREAP_v0.2 software. These novel miRNAs are shown in Additional file 2.

### Different expression analysis of miRNAs

**Fig. 4:**
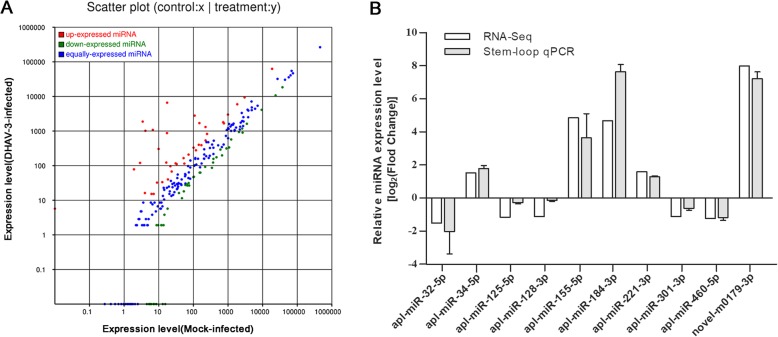
Comparison of miRNA expression between the mock-infected and DHAV-3-infected libraries. **a** The scatter plot of differentially expressed miRNAs of DHAV-3-infected vs. mock-infected libraries. **b** Validation of DEMs by stem-loop quantitative RT-PCR. The relative expression level of each miRNA in DHAV-3-infected sample was calculated using the 2^−ΔΔCt^ method and represented as an n-fold change compared to the mock-infected sample

For expression comparison between DHAV-3-infected and mock-infected libraries, miRNA sequences were analyzed through log2 (ratio) test and Chi-square 2 × 2 tests based on their normalized reads. Following significant differences standard (*p* < 0.05 and |log2 (fold change)| ≥ 1), 156 differentially expressed miRNAs (DEMs) were detected in the two libraries (Fig. 4a, Additional file 3). Compared to the mock-infected library, 102 miRNAs were upregulated and 54 miRNAs were downregulated in the DHAV-3-infected library (Fig. 4a, Additional file 3).

### Target genes prediction for miRNAs

In order to understand the molecular function and biological processes of miRNAs during DHAV-3 infection in duckling liver, three independent algorithms, RNAhybrid, Miranda and TargetScan were used to predict the mRNA targets. A total of 26,886 genes for 398 known miRNAs and 119 novel miRNAs were predicted as potential miRNA targets. GO analysis revealed that these predicted target genes were involved in biological process, cellular component and molecular function (Additional file 4). To explore the roles that DEMs may play in regulatory networks, we also assigned the putative miRNA targets to the KEGG pathways using the KEGG Orthology Based Annotation System (KOBAS). The results showed that 6746 candidate genes were annotated for 241 biological processes. Many immune-related pathways, such as apoptosis (ko04210), ubiquitin mediated proteolysis (ko04120), FoxO signaling pathway (ko04068), NOD-like receptor signaling pathway (ko04621), p53 signaling pathway (ko04115), and RIG-I-like receptor signaling pathway (ko04622), were significantly enriched (Additional file 5). The results indicated that DEMs might play an important role in the virus-host interactions during the DHAV-3 infection in duck.

Furthermore, 23 DEMs, including 18 known miRNAs and 5 novel miRNAs, were predicted to target the DHAV-3 genome (Additional file 6). Most of these miRNAs were predicted to target genes of DHAV-3 structural proteins. For example, the VP1 gene of DHAV-3 was predicted to be targeted by 7 miRNAs, the VP0 gene was targeted by 5 miRNAs, and the VP3 gene was targeted by 3 miRNAs. Moreover, 4 non-structural protein genes of DHAV-3, including the 2C, 3B, 3C and 3D genes, were predicted targets of 7 DEMs. In addition, the apl-miR-340-5p was predicted to target the 5′-untranslated region (5′-UTR) of the DHAV-3 genome.

### Global transcriptome profiles

In parallel with the miRNA profile, we also explored the global changes in gene expression associated with DHAV-3 infection to assess the effect of DEMs on their predicted targets. Six cDNA libraries representing the livers of ducklings in the mock-infected group (C1, C2, C3) and those in the DHAV-3-infected group (SD1, SD2, SD3) were constructed and subjected to Illumina sequencing. An overview of the sequencing data is shown in Additional file 7. Q20 and Q30 values were all > 95%, and GC content was similar, indicating that the data was accurate and reliable. Approximately 70% of the clean reads mapped to the duck reference genome (CAU_duck1.0) (Table 2).

**Table 2:** Major characteristics of mRNA libraries and reads mapping to the duck reference genome

Sample	Clean reads	Filter reads	Reads mapping to genome			
			Unique mapped reads	Mutiple mapped reads	Unmapped reads	Mapping ratio (%)
C1	71,247,742	68,240,188	51,250,772	258,906	16,730,510	75.48
C2	55,513,732	53,236,588	39,110,688	254,606	13,871,294	73.94
C3	66,268,876	63,854,884	47,976,494	280,978	15,597,412	75.57
SD1	58,962,100	54,740,070	38,301,958	154,838	16,283,274	70.25
SD2	56,170,156	52,753,656	36,074,767	138,914	16,539,975	68.65
SD3	52,572,684	50,532,548	35,338,685	166,060	15,027,803	70.26

### Analysis of DEGs

**Fig. 5:**
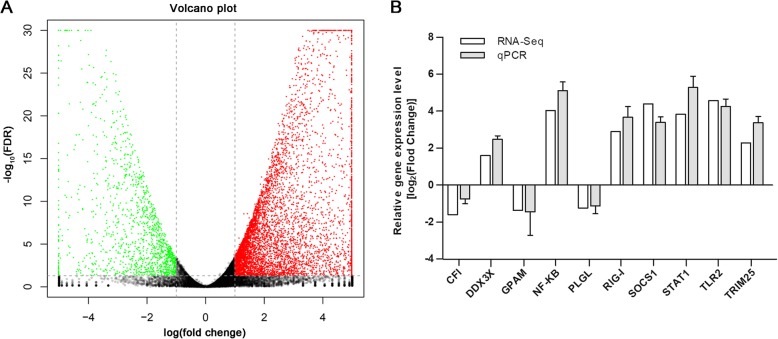
Comparison of gene expression between the mock-infected and DHAV-3-infected libraries. **a** The volcano plot of differentially expressed genes of DHAV-3-infected vs. mock-infected libraries. Red points represent up-regulated genes, green points represent down-regulated genes, and black points represent genes with no significant difference. **b** Validation of DEGs by qRT-PCR. The relative expression level of each gene in DHAV-3-infected sample was calculated using the 2^−ΔΔCt^ method and represented as the n-fold change compared to the mock-infected sample

Genes with at least a twofold change (| log2 (fold change) | ≥ 1) and the FDR < 0.05 were considered DEGs. As a result, a total of 7717 DEGs, including 6358 up-expressed and 1359 down-expressed, were generated in DHAV-3-infected duckling livers compared with those in the mock-infected ducklings at 24 hpi (Fig. 5a and Additional file 8). It is notable that there are more up-regulated genes than down-regulated genes at 24 hpi. Overall, DHAV-3 infection had a significant impact on the global gene expression profile.

To determine functionality of the DEGs, we performed GO enrichment analysis (Additional file 9). The DEGs were mainly enriched in 244 biological processes, mainly including the regulation of immune system process (GO:0002682), regulation of response to stimulus (GO:0048583), and lymphocyte activation (GO:0046649), of which the immune-related GO term accounted for the most enriched terms (Additional file 10).

KEGG pathway enrichment analysis was used to further define DEGs function in duckling liver after DHAV-3 infection (Additional file 11). The top 20 significantly enriched KEGG pathways are listed in Additional file 12 based on a *q*-value < 0.05. Five functional categories were identified to potentially play a role in DHAV-3 infection, including cytokine-cytokine receptor interaction (ko04060), Jak-STAT signaling pathway (ko04630), Toll-like receptor signaling pathway (ko04620), Influenza A (ko05164), and apoptosis (ko04210). These results suggested that genes in these pathways may be involved in response to DHAV-3 infection in ducklings.

### Integrated analysis of DEMs and DEGs

The correlation between miRNAs and mRNAs was based on the miRNA and transcriptome sequencing. To construct miRNA-mRNA networks, the genes identified as putative targets of DEMs, which were also differentially expressed in the transcriptome, were selected as the candidate target genes. According to the negative correlation principle of miRNA and mRNA, we anticipated an inverse relationship between the miRNAs and their target genes. Based on these criteria, 19,606 miRNA-mRNA interactions with the involvement of 155 DEMs and 4484 DEGs were identified (Additional file 13).

**Fig. 6:**
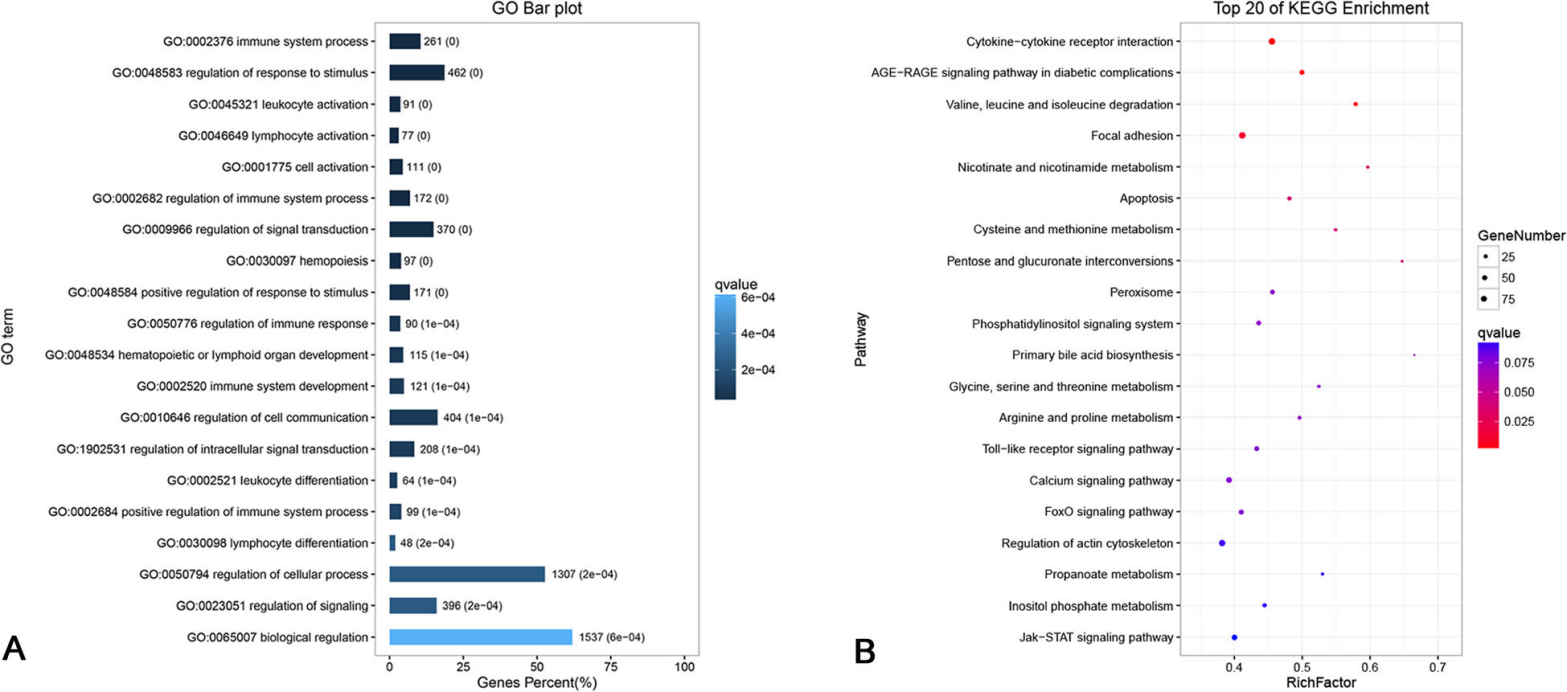
Functional enrichment analysis of genes in the negatively correlated miRNA-mRNA network. **a** The top 20 enriched GO biological processes of genes involved in the negative miRNA-mRNA network. The darker the color of the bar, the smaller the q-value. The numbers indicate the related gene number and the q-value of each GO term. **b** The top 20 enriched KEGG pathways of genes involved in the negative miRNA-mRNA network. The larger the dot, the greater the number of genes. The redder the color, the smaller the q-value

**Fig. 7:**
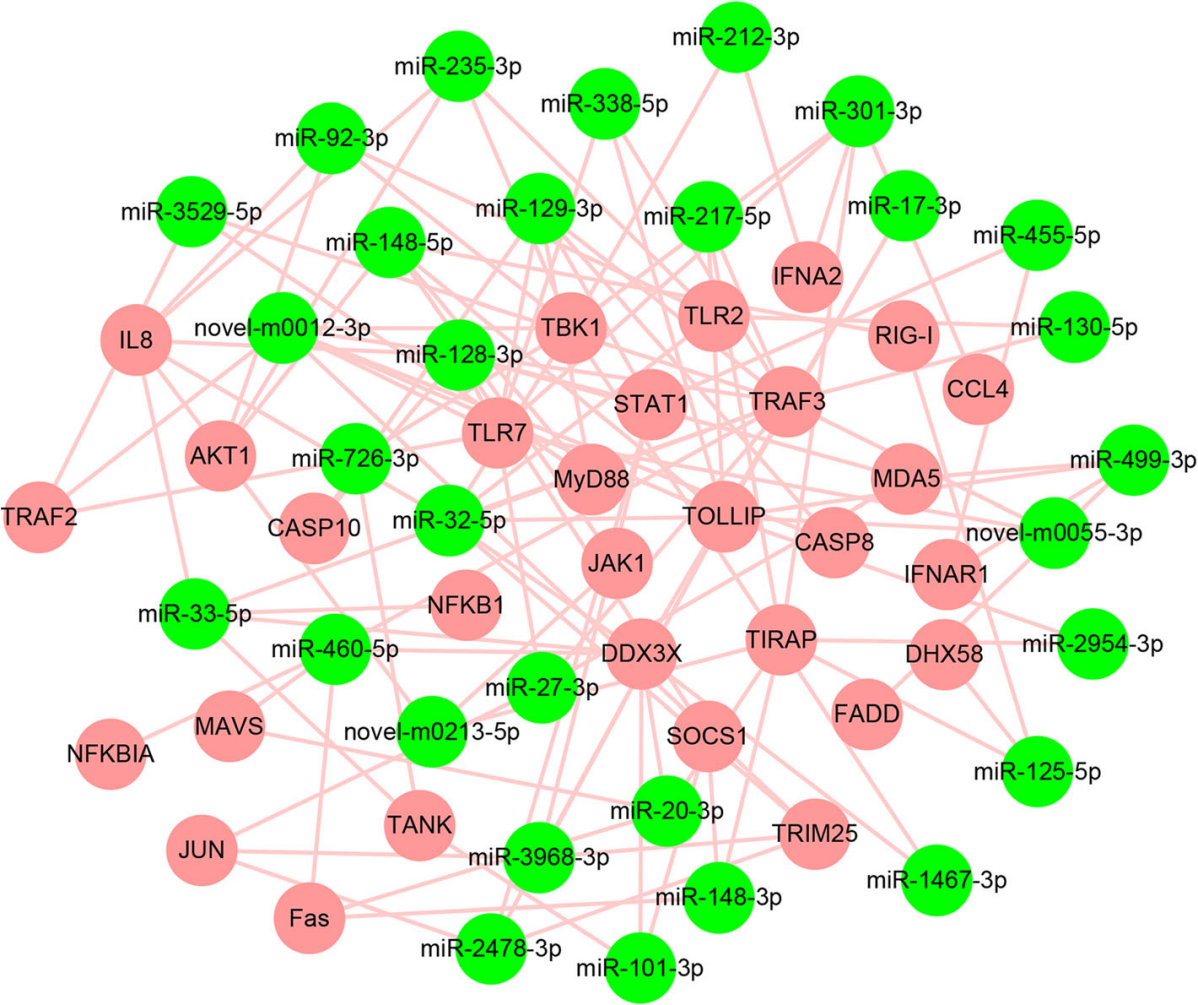
Immune-related miRNA-mRNA negative correlation network. The interaction network constructed on the basis of immune-related DEMs and DEGs of DHAV-3-infected vs. mock-infected comparisons. The miRNAs are displayed as green circles, and the target genes are shown as pink circles

Functional enrichment analysis of genes in these negatively correlated miRNA-mRNA pairs provided us with an integrated picture of their functional roles during DHAV-3 infection in ducklings. Among the top 20 significantly enriched GO terms in biological process, immune- and signal-related terms were in the majority (Fig. 6a). Pathway analysis help us to obtain a better understanding of the biological function of genes. The KEGG pathway analysis demonstrated that the target genes of 155 DEMs were significantly associated with cytokine-cytokine receptor interaction (ko04060), apoptosis (ko04210), Toll-like receptor signaling pathway (ko04620), FoxO signaling pathway (ko04068), and Jak-STAT signaling pathway (ko04630) (Fig. 6b). A partial miRNA-mRNA interaction network of these pathways associated with DHAV-3 and host interactions were generated in Fig. 7, using Cytoscape network construction software.

### Quantitative RT-PCR validation of significant DEMs and DEGs

Quantitative RT-PCR was performed to investigate the differentially expressed miRNAs and mRNAs from sequencing data. Ten DEMs (including nine known miRNAs and a novel candidate miRNA) and 10 DEGs were randomly selected for validation. Overall, the results analyzed by qPCR were in accordance with the high-throughput sequencing data (Fig. 4b and Fig. 5b). This confirmed the reliability of the sequencing techniques used in this work.

## Discussion

High-throughput sequencing is a powerful tool to reveal the expression profiles of miRNAs and genes in animals and plants. It is especially useful for identifying differentially expressed miRNAs and genes under physiological perturbation. The combination of the miRNA profile with transcriptome has great significance to the study of miRNA regulatory mechanisms during the virus infection process. Although some researchers had investigated changes of the miRNA expression profile during the process of some viruses infected duck cells [27, 28], to our knowledge, the miRNA expression profile in ducklings exposed to DHAV-3 has not been identified. To this end, in the present study high-throughput sequencing was conducted to identify miRNAs and genes involved in the response to DHAV-3 in duckling liver. We also attempted to construct a miRNA-mRNA regulatory network according to the DEMs and DEGs datasets. Differential expression of 156 miRNAs and 7717 mRNAs with 19,606 negative miRNA-mRNA pairs in DHAV-3-infected duckling liver were identified successfully. As shown in the miRNA-mRNA regulatory network (Fig. 7), a single miRNA can target multiple genes. For example, apl-miR-125-5p can synchronously target RIG-I (retinoic acid inducible gene-I), DHX58 (DEXH box polypeptide 58), and TIRAP (toll/interleukin-1 receptor domain-containing adapter protein). On the contrary, one gene can be targeted by several miRNAs, such as SOCS1 (suppressor of cytokine signaling 1) which can be simultaneously targeted by apl-miR-101-3p, apl-miR-128-3p, and apl-miR-148-3p (Fig. 7). However, the network showed in Fig. 7 is only a small part of the entire network. The actual miRNA-mRNA regulatory network is much more complex.

MicroRNA-mediated changes in gene expression have been demonstrated to modulate viral replication, antiviral immune responses, viral latency, and pathogenesis [18, 29, 30]. In order to highlight the miRNAs related to interactions between DHAV-3 and ducklings, we employed pathway analysis of mRNAs in the negative miRNA-mRNA network to research their biological functions and molecular mechanisms. In KEGG pathway analysis for 4484 mRNAs involved in the negative miRNA-mRNA network, immune-related pathways, including cytokine-cytokine receptor interaction, apoptosis, Toll-like receptor, FoxO, Jak-STAT, and RIG-I-like receptor signaling pathways were significantly enriched. Similarly, pathway enrichment analysis for 7717 DEGs of the transcriptome produced similar results. This pathway enrichment result is also consistent with another study that investigated the transcriptome of DHAV-3 infected ducklings at 48 hpi [9]. Previous reports have shown that these pathways are involved in the host anti-virus processes [31–34]. Briefly, miRNAs influence the regulation of immune responses and may be related to DHAV-3 replication at the early infection stage in ducklings.

The innate immune system is the first line of defense against invading viruses [35, 36]. To date, accumulating data have proven a particular role for miRNAs in modulating many levels of the innate immune response [35, 37]. Pattern-recognition receptors (PRRs), such as Toll-like receptors (TLRs) and RIG-I-like receptors, recognize pathogens and initiate a series of signaling processes that execute the first line of host innate immune responses. In our study, the transcriptional expressions of TLR2 and TLR7 were significantly up-regulated. Meanwhile, the negative miRNA-mRNA network revealed 12 significantly down-regulated miRNAs targeting these TLRs. It has been reported that some miRNAs, like miR-140-5p and well-described miR-155, could target TLRs to regulate the innate immune response during the virus infection that had subsequent impact on virus replication [38–40]. Upon recognition of pathogens, TLRs recruit a specific set of adaptor molecules, such as MyD88 (myeloid differentiation factor 88) and TIRAP, then initiate downstream signaling events that leads to the secretion of inflammatory cytokines, type I IFN, chemokines, and antimicrobial peptides [41]. In the present study, we found that apl-miR-129-3p, apl-miR-148-5p, and apl-miR-3529-5p were down regulated upon DHAV-3 infection, and they all targeted MyD88 (Fig. 7). In addition, significantly up-regulated TIRAP after DHAV-3 infection was targeted by 7 miRNAs in our network (Fig. 7). MyD88 was targeted by miR-7 and miR-21 during several viruses infection enabling these viruses to evade the immune surveillance system to enhance their proliferation [42–44]. It is important to note that, apl-miR-32-5p has multiple target genes in the Toll-like receptor signaling pathway, including TLR2, TOLLP (toll-interacting protein), TRAF3 (TNF receptor-associated factor 3), and TBK1 (TANK-binding kinase 1). Similarly, apl-miR-301-3p had multiple target genes (targeting TLR7, TIRAP, TBK1, and CCL4) as did novel-m0012-3p (targeting TLR7, TOLLP, TRAF3, and TBK1). This indicates that these three miRNAs may play a regulative role in the Toll-like receptor signaling pathway.

In the RIG-I-like receptor signaling pathway, the downregulated apl-miR-125-5p and apl-miR-148-5p were observed to bring about the upregulation of RIG-I in the DHAV-3 infected liver of ducklings. RIG-I recognizes viral RNA in the cytosol of most cell types that executes a critical role in detection of RNA viruses [32]. Similarly, the melanoma differentiation-associated gene 5 (MDA5) which is another virus RNA detector was targeted by apl-miR-128-3p, apl-miR-27-3p, and apl-miR-499-3p. There are researches demonstrated that gga-miR-142-5p and miR-34b-5p could target MDA5 to promote IBDV (infectious bursal disease virus) and ALV-J (avian leukosis virus subgroup J) replication, respectively [45, 46]. In our miRNA-mRNA network, four miRNAs (e.g. apl-miR-32-5p, novel-m0012-3p) targeted the RIG-I signaling-related molecule TIRM25 (tripartite motif containing 25). Moreover, MAVS (mitochondrial antiviral signaling protein), which functions as a platform for innate antiviral signal transduction and positively regulates type I interferon (IFN) production, was targeted by apl-miR-20-3p and apl-miR-460-5p (Fig. 7). Detailed analysis revealed that miRNAs suppressed the expression of MAVS, thereby inhibiting MAVS-mediated NF-kappa B and IRF3 signaling, decreasing type I IFN and antiviral gene production, thus facilitating viral replication [47]. Briefly, DEGs with their target miRNAs in the RIG-I-like receptor signaling pathway may have important roles in regulating the innate immune system during DHAV-3 infection.

IFNs provide a robust line of host innate immune defense against viral infection [48]. IFN-α and IFN-β expressions can be induced in the DHAV-3 infected duckling liver [10]. However, little is known about the mechanisms for miRNA regulation of IFN induction in DHAV-3 infection in ducklings. At the type I IFN receptor level, multiple miRNAs can target the interferon alpha/beta receptor (IFNAR) to regulate type I IFN signaling [35, 49, 50]. In the current study, the expression of IFNAR1 and IFNAR2 had a 2.15-fold and a 4.88-fold increase, respectively. Apl-miR-455-5p and apl-miR-499-3p which targeted IFNAR1, plus novel-m0213-5p which targeted IFNAR2 were significantly down-regulated after DHAV-3 infection. Moreover, in our negative miRNA-mRNA network, five miRNAs (e.g. apl-miR-128-3p, apl-miR-455-5p) targeted the signal transducer and activator of transcription 1 (STAT1) which is a member of JAK-STAT pathway. As a transcription factor, STAT1 plays an important role in antiviral responses by inducing expression of antiviral IFN-stimulated genes (ISGs) [51]. Hou et al. found that miR146a targeted STAT1 and attenuated the production of type I IFN-induced antiviral factors [52]. Also, miRNAs can indirectly modulate type I IFN signal transduction by targeting SOCS1, a negative regulator of the JAK-STAT pathway [53]. We found that the negative regulator SOCS1 was targeted by apl-miR-101-3p, apl-miR-128-3p and apl-miR-148-3p in DHAV-3-infected duckling livers. These results indicate that miRNAs are involved in many stages of the IFN response, such as by regulation of their cognate receptor components IFNAR and downstream signal transduction pathways including STATs. This may give an insight into the post-transcriptional gene regulation mechanisms of IFN signaling in DHAV-3 infection.

It is important to note that several miRNAs discussed above, such as apl-miR-32-5p, apl-miR-125-5p, apl-miR-128-3p, apl-miR-460-5p, and novel-m0012-3p, can target vital genes that are not only involved in the Toll-like receptor and the RIG-I-like receptor signaling pathway, but also involved in their downstream signaling pathways (Table 3). These results indicate the possibility that these miRNAs are vital regulators participating in the immune-related signaling pathways in DHAV-3-infected ducklings. And further investigations are needed to clarify the underlying mechanisms by which these miRNAs regulate their target genes and their roles in promoting or inhibiting DHAV-3 replication.

**Table 3:** Candidate immune-related miRNAs in the DHAV-3-infected duckling

miRNA	Up down regulation	*p*-value	Candidate target gene	Pathway
apl-miR-32-5p	Down	4.40E-84	*TRIM25*	RIG-I-like receptor signaling pathway
			*DDX3X*	RIG-I-like receptor signaling pathway
			*TLR2*	Toll-like receptor signaling pathway
			*TOLLIP*	Toll-like receptor signaling pathway
			*TRAF3*	Toll-like receptor signaling pathway; RIG-I-like receptor signaling pathway
			*TBK1*	Toll-like receptor signaling pathway; RIG-I-like receptor signaling pathway
			*IL8*	Cytokine-cytokine receptor interaction; Toll-like receptor signaling pathway; RIG-I-like receptor signaling pathway; NOD-like receptor signaling pathway
apl-miR-125-5p	Down	5.18E-113	*RIG-I*	RIG-I-like receptor signaling pathway
			*DHX58*	RIG-I-like receptor signaling pathway
			*TIRAP*	Toll-like receptor signaling pathway
apl-miR-128-3p	Down	1.35E-128	*MDA5*	RIG-I-like receptor signaling pathway
			*TLR7*	Toll-like receptor signaling pathway
			*CASP10*	RIG-I-like receptor signaling pathway; Apoptosis
			*STAT1*	Jak-STAT signaling pathway
			*SOCS1*	Jak-STAT signaling pathway
			*IL8*	Cytokine-cytokine receptor interaction; Toll-like receptor signaling pathway; RIG-I-like receptor signaling pathway; NOD-like receptor signaling pathway
apl-miR-460-5p	Down	2.39E-16	*MAVS*	RIG-I-like receptor signaling pathway
			*DDX3X*	RIG-I-like receptor signaling pathway
			*TRAF3*	Toll-like receptor signaling pathway; RIG-I-like receptor signaling pathway
			*Fas*	Cytokine-cytokine receptor interaction; MAPK signaling pathway; Apoptosis; p53 signaling pathway
			*NFKBIA*	Toll-like receptor signaling pathway; RIG-I-like receptor signaling pathway; NOD-like receptor signaling pathway; Apoptosis
novel-m0012-3p	Down	3.91E-02	*TRIM25*	RIG-I-like receptor signaling pathway
			*TRAF2*	RIG-I-like receptor signaling pathway; MAPK signaling pathway
			*TLR7*	Toll-like receptor signaling pathway
			*TOLLIP*	Toll-like receptor signaling pathway
			*TRAF3*	Toll-like receptor signaling pathway; RIG-I-like receptor signaling pathway
			*TBK1*	Toll-like receptor signaling pathway; RIG-I-like receptor signaling pathway
			*CASP8*	Toll-like receptor signaling pathway; RIG-I-like receptor signaling pathway; NOD-like receptor signaling pathway; Apoptosis; p53 signaling pathway

Evidence is accumulating that host miRNAs can bind to a broad range of RNA viruses, directly regulating their pathogenesis [18]. The outcome of these interactions, that the viral replication is directly altered, have been identified [54, 55]. Interestingly, we predicted that some miRNAs target genes of the DHAV-3 genome in this study. Considering the ability of RNA viruses to rapidly alter their genomes in the face of selective pressure [56], the binding of these candidate miRNAs to the DHAV-3 RNA and the interaction between these candidate miRNAs and the viral RNA requires further validation.

## Conclusions

This study is the first exploration to simultaneously characterize miRNAs and mRNAs of ducklings in response to DHAV-3 infection. Numerous differentially expressed miRNAs and mRNAs were screened and identified. According to our data of integrated analysis, a negative miRNA-mRNA network was constructed. The functional enrichment analysis of genes involved in the network provided information which could help us explain the host-virus interactions during DHAV-3 infection. To resist viral infection, the ducklings mobilized many immune-related miRNAs and genes in several pathways, such as Toll-like receptor signaling pathway, RIG-I-like receptor signaling pathway, and Jak-STAT signaling pathway. We also found that some miRNAs (e.g. apl-miR-32-5p, apl-miR-125-5p, apl-miR-128-3p, apl-miR-460-5p, and novel-m0012-3p) may play an important role in regulating the host defense response and effecting DHAV-3 replication. In addition, some host miRNAs were predicted to target the DHAV-3 genome. We believe that these data will contribute to further studying the pathogenic mechanism of DHAV-3.

## Methods

### Virus and animals

Twenty-five specific-pathogen-free (SPF) duck embryos were obtained from the Laboratory Animal Center in the Harbin Veterinary Research Institute at the Chinese Academy of Agricultural Sciences (HVRI; Harbin, China). Twenty SPF ducklings used in this study were hatched from these SPF duck embryos and housed in isolators. All animal experiments were approved by the Animal Ethics Committee of Jiangsu Academy of Agricultural Sciences (approval number SYXK-Su-2015-0020) and followed the Regulation on the Administration of Laboratory Animals (2017 Revision, Ministry of Science and Technology of the People’s Republic of China).

The highly virulent DHAV-3 SD strain used in this study was isolated in Shandong Province by our own laboratory. The virus was propagated in 1- to 3-day-old SPF ducklings. The virus titer was calculated by the Reed and Muench method and determined to be a 10^6.3^ lethal median dose (LD_50_)/mL.

### DHAV-3 infection and sample collection

Twenty 1-day-old SPF ducklings were randomly chosen and intramuscularly (IM) inoculated with 0.2 mL of the lethal DHAV-3 SD strain under 10^5^ dilutions. At 3, 6, 12, 24 and 36 h post-infection (hpi), three infected ducklings were euthanized by decapitation. Their liver tissues were collected, washed with ice-cold PBS, frozen in liquid nitrogen immediately, then stored at − 80 °C until the used for total RNA extraction for qRT-PCR, transcriptome and miRNA sequencing analysis.

### RNA isolation and virus loads detection

Total RNA of all samples was isolated using TRIzol® reagent (Invitrogen, Carlsbad, CA, USA) following the manufacturer’s instruction. The quantity and quality of the extracted total RNAs were detected and assessed using a NanoDrop 2000 spectrophotometer (Thermo Fisher Scientific, Waltham, MA, USA) and an Agilent 2100 RNA 6000 Nano Kit (Agilent Technologies, Santa Clara, CA, USA).

Viral copies in total RNA were measured using TaqMan-based RT-qPCR established in our laboratory. The qPCR was carried out on an ABI Step One Plus thermocycler (Applied Biosystems, Foster City, CA, USA) with a final volume of 20 μL using the One Step PrimeScript RT-PCR Kit (TaKaRa, Dalian, China) according to the manufacturer’s instructions. The probe and primer are listed in Additional file 14. Their final concentrations were 0.4 μM and 0.2 μM respectively. In addition, the amplicon of the forward and reverse primer pair was cloned into the pMD18-T vector (TaKaRa, Dalian, China) using as the DHAV-3 standard plasmid for constructing the standard curve of virus copy. Thermal cycling conditions included 42 °C for 5 min, 95 °C for 10 s, 40 cycles of 95 °C for 5 s and 60 °C for 34 s. Negative controls contained PCR-grade water. All reactions were conducted in triplicate.

### Small RNA sequencing and data analysis

Small RNA (sRNA) libraries were generated from DHAV-3-infected and mock-infected duckling livers at 24 hpi. The sequencing of miRNAs was performed by Gene Denovo Biotechnology Co. (Guangzhou, China) using Illumina HiSeq™ 2500. The sequencing data analysis pipeline is outlined in Fig. 1a. The raw reads were first trimmed of adapters and low quality bases to get clean tags. Then clean tags were mapped to GenBank database (Release 209.0) and Rfam database (14.0) via blastall 2.2.25 (blastn, with the parameter identity > 97%) to identify and remove rRNA, scRNA, snoRNA, snRNA and tRNA. The clean tags were also aligned with the reference duck genome (CAU_duck1.0) by Bowtie (version 1.1.2, with the main parameters -v 0 --best --strata -a) [57]. Those mapped to exons, introns, and repeat sequences were removed. After the sRNAs screened using the above processes were excluded, the remaining sRNAs were searched against the precursor miRNAs in the miRBase database (Release 22.0) via Bowtie (version 1.1.2, with the main parameters -v 2 --best --strata -a) to identify known miRNAs. For prediction of novel miRNAs, all of the unannotated tags were aligned with the reference genome in the same way as above. Then the hairpin structures of those matched tags were predicted by software Mireap_v0.2 [58] (with parameters -A 18, −B 26, −a 20, −b 24, −c 3, −u 20, −e − 18, −d 35, −p 14, −v 4, −s 5, −f 10) to identify the novel miRNA candidates.

To profile the differentially expressed miRNAs in DHAV-3-infected libraries versus mock-infected libraries, the miRNA expression level was calculated and normalized to transcripts per million (TPM). The *p*-value was adjusted using the Benjamini-Hochberg method [59, 60]. A corrected *p*-value < 0.05 and | log2 (fold change) | ≥ 1 were set as the threshold parameters for the significant DEMs. Miranda (v3.3a) [61], TargetScan (Version 7.0) [62] and RNAhybrid (v2.1.2) + svm_light (v6.01) [63] were used to predict the target genes. Then the intersection set of the results from the three software programs was chosen as the predicted miRNA target genes.

### Transcriptome sequencing and data analysis

The RNA samples used for the sRNA sequencing were the same as those used for the transcriptome sequencing. After total RNA extraction and DNase I treatment, mRNA was enriched by Oligo(dT) beads. Then the enriched mRNA was fragmented into short fragments using fragmentation buffer, and the reverse transcript of mRNA was completed with random primers. Second-strand cDNA were synthesized. The cDNA fragments were purified, end repaired, poly(A) added, and ligated to Illumina sequencing adapters. The ligation products were size selected by agarose gel electrophoresis, PCR amplified, and sequenced using Illumina HiSeq™ 2500 by Gene Denovo Biotechnology Co. (Guangzhou, China). The pipeline of transcriptome data analysis is shown in Fig. 1b. Raw sequence data were assessed and filtered. Clean reads were aligned to the reference duck genome (CAU_duck1.0). The reconstruction of transcripts was carried out with Cufflinks software [64]. The expression of each gene was calculated according to the reads per kilo bases per million reads (RPKM). To identify DEGs, the edgeR package (http://www.rproject.org/) was used. In addition, | log2 (fold change) | ≥ 1 and the false discovery rate (FDR) < 0.05 were used as thresholds to define significant differences in gene expression.

### Construction of miRNA-mRNA regulatory networks

To elucidate the interaction network of miRNA-mRNA with positive and negative correlations, the DEMs and DEGs were used to construct a miRNA-mRNA regulatory network (Fig. 1c). Expression correlation between miRNA-mRNA was evaluated using the Pearson correlation coefficient (PCC). Pairs with PCC < − 0.7 and *p* < 0.05 were selected as co-expressed negatively miRNA-mRNA pairs. Visualization of the miRNA-mRNA network was conducted using Cytoscape software (v3.6.0).

### Functional analysis

To assess functional enrichment, Gene ontology (GO) functional analysis and a Kyoto Encyclopedia of Genes Genomes (KEGG) pathways analysis against the mRNAs in the network were implemented [65, 66]. The significantly enriched GO or KEGG terms were analyzed using *p*-value < 0.05.

### Quantitative RT-PCR verification

The RNA samples used for the high-throughput sequencing assays were also used for the qPCR assay. The DEMs were validated by stem-loop qRT-PCR [67, 68]. Briefly, 1 μg of total RNA was reverse-transcribed to cDNA. The cDNA was synthesized with stem-loop reverse-transcribed primers for DEMs validation, using PrimeScript™ RT Master Mix (TaKaRa, Dalian, China). The cDNA was synthesized with oligo(dT) primer for DEGs validation, using ReverTra Ace® qPCR RT Kit (TOYOBO, Osaka, Japan). Next, 2 μL cDNA was used for qPCR according to PowerUp™ SYBR® Green Master Mix kit (Applied Biosystems, Foster City, CA, USA) instructions. All primers are given in Additional file 14. The small nuclear RNA (snRNA) U6 was used as endogenous internal control gene for miRNA and GAPDH was used for mRNA. ABI Step One Plus thermocycler (Applied Biosystems, CA, USA) was used to conduct qRT-PCR. Reactions were performed in triplicate, and the relative expression levels were quantified by the 2^-ΔΔCt^ method [69].

## Supplementary information

**Additional file 1:** Known miRNAs identified in this study.

**Additional file 2:** Novel miRNAs identified in this study.

**Additional file 3:** Differentially expressed miRNAs in mock-infected and DHAV-3-infected duckling liver.

**Additional file 4:** GO annotation of the predicted target genes of all miRNAs in this study.

**Additional file 5:** KEGG enrichment analysis of target genes annotated for miRNAs differentially expressed in mock- and DHAV-3-infected duckling liver.

**Additional file 6:** Differentially expressed miRNAs predicted to target the DHAV-3 genome.

**Additional file 7:** Overview of mRNA-Seq in the mock-infected and DHAV-3-infected libraries.

**Additional file 8:** Differentially expressed genes in mock-infected and DHAV-3-infected duckling liver.

**Additional file 9:** GO enrichment analysis of DEGs in mock-infected and DHAV-3-infected duckling liver.

**Additional file 10:** GO analysis information of DEGs significantly enriched in 244 biological processes.

**Additional file 11:** KEGG pathway analysis of DEGs in mock- and DHAV-3-infected duckling livers.

**Additional file 12:** The top 20 enriched KEGG pathways of DEGs.

**Additional file 13:** The list of 155 DEMs and their target genes in the negative miRNA-mRNA network.

**Additional file 14:** The primers used in this study.

## Data Availability

The Illumina sequencing data from this study have been submitted to NCBI Sequence Read Archive under the accession number SRP220254, which is associated with BioProject number PRJNA563502 (https://trace.ncbi.nlm.nih.gov/Traces/sra/?study=SRP220254). Raw reads for small RNA sequencing data: SRX6792052 and SRX679205. Raw reads for transcriptome sequencing data: SRX6792046, SRX6792047, SRX6792048, SRX6792049, SRX6792050, and SRX6792051. The datasets supporting the conclusions of this article are included within the article and additional files.

## Abbreviations

3′-UTR: 3′-untranslated region
ALV-J: Avian leukosis virus subgroup J
DEGs: Differentially expressed miRNAs
DEMs: Differentially expressed miRNAs
DENV: Dengue virus
DHAV-3: Duck hepatitis A virus type 3
DHX58: DEXH box polypeptide 58
GO: Gene ontology
hpi: Hour post-infection
IBDV: Infectious bursal disease virus
IFN: Interferon
IFNAR: Interferon alpha/beta receptor
ISGs: IFN-stimulated genes
JEV: Japanese encephalitis virus
KEGG: Kyoto Encyclopedia of Genes Genomes
MAVS: Mitochondrial antiviral signaling protein
miRNAs: MicroRNAs
MyD88: Myeloid differentiation factor 88
PCC: Pearson correlation coefficient
PRRs: Pattern-recognition receptors
RIG-I: Retinoic acid inducible gene-I
snRNA: small nuclear RNA
SOCS1: Suppressor of cytokine signaling 1
SPF: Specific-pathogen-free
sRNA: Small RNA
STAT1: Signal transducer and activator of transcription 1
TBK1: TANK-binding kinase 1
TIRAP: Toll/interleukin-1 receptor domain-containing adapter protein
TIRM25: Tripartite motif containing 25
TLRs: Toll-like receptors
TOLLP: Toll-interacting protein
TPM: Transcripts per million
TRAF3: TNF receptor-associated factor 3
WNV: West Nile virus
