# Supplementary figures and images for: Immune-related miRNA-mRNA regulation network in the livers of DHAV-3-infected ducklings
Source: BMC Genomics. 2020 Feb 4;21:123. doi: 10.1186/s12864-020-6539-7 (PMC7001231; doi:10.1186/s12864-020-6539-7)

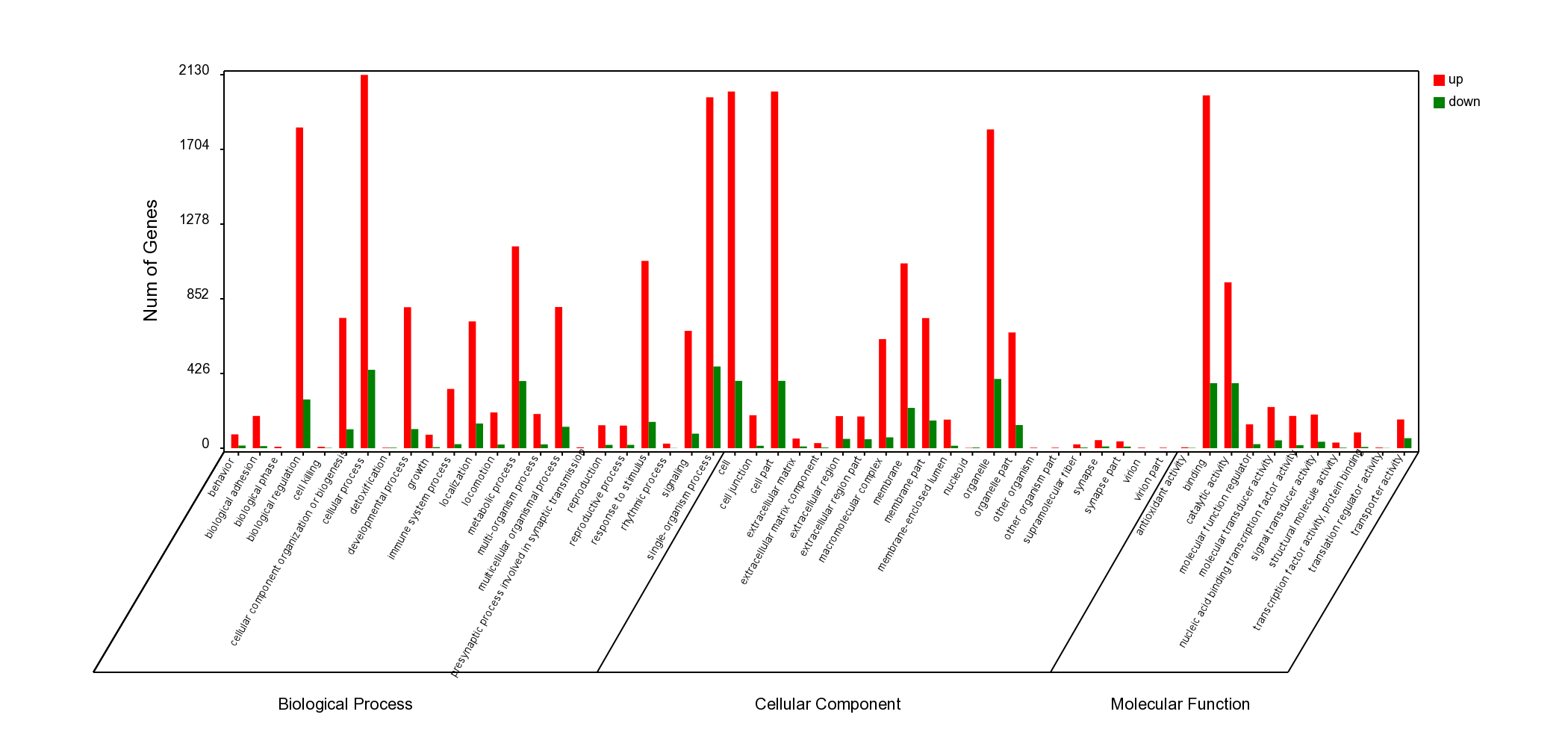

Supplement: Supplementary file 9 — Additional file 9: GO enrichment analysis of DEGs in mock-infected and DHAV-3-infected duckling liver. [file 12864_2020_6539_MOESM9_ESM.png]

# Top 20 of Pathway Enrichment

Pathway

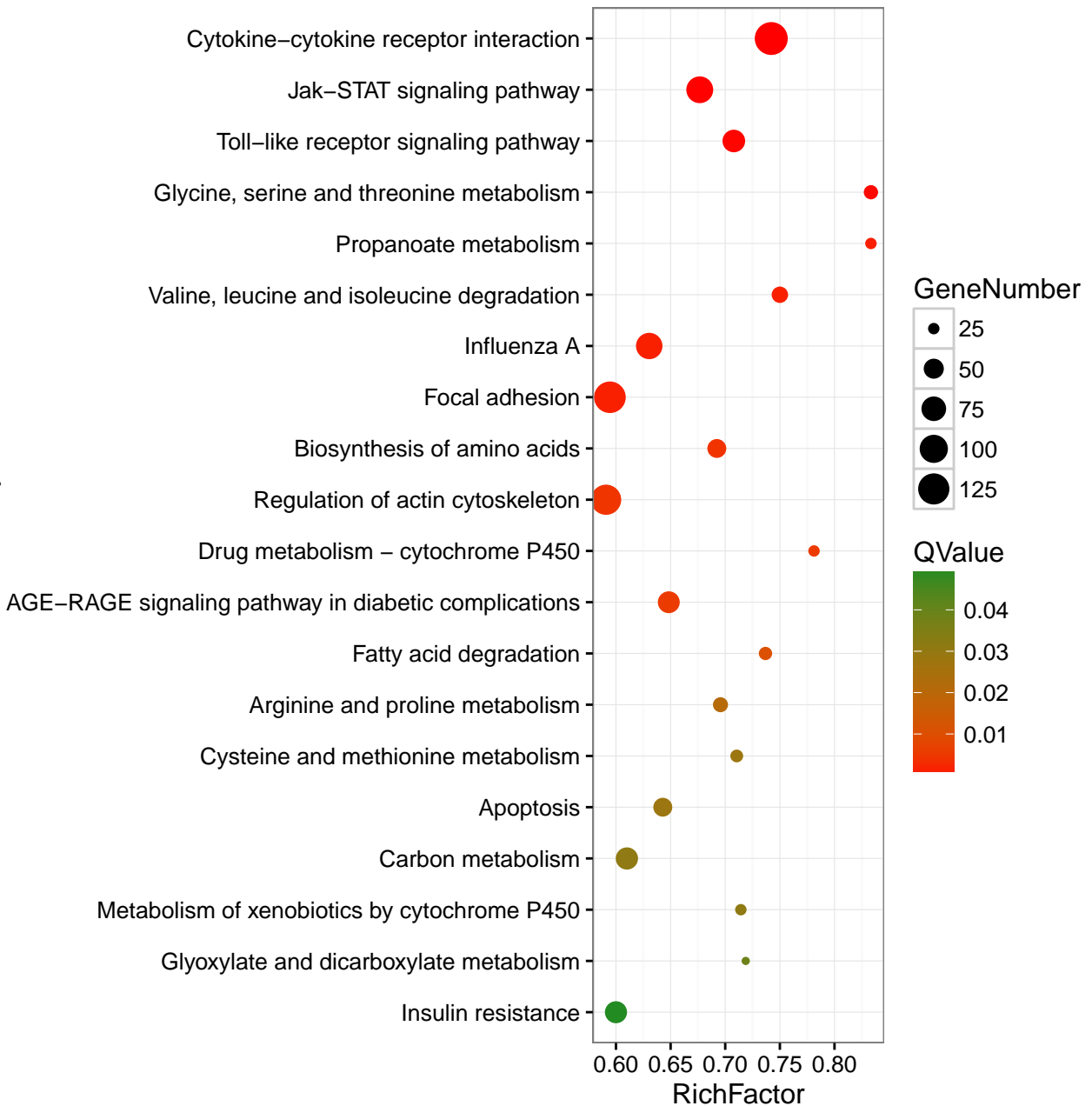

Supplement: Supplementary file 12 — Additional file 12: The top 20 enriched KEGG pathways of DEGs. [file 12864_2020_6539_MOESM12_ESM.pdf]
